# Cumulative Effects of Neighborhood Social Adversity and Personal Crime Victimization on Adolescent Psychotic Experiences

**DOI:** 10.1093/schbul/sbx060

**Published:** 2017-05-22

**Authors:** Joanne Newbury, Louise Arseneault, Avshalom Caspi, Terrie E Moffitt, Candice L Odgers, Helen L Fisher

**Affiliations:** 1 MRC Social, Genetic & Developmental Psychiatry Centre, Institute of Psychiatry, Psychology & Neuroscience, King’s College London, London, UK; 2 Departments of Psychology and Neuroscience, Psychiatry and Behavioral Sciences, and Centre for Genomic and Computational Biology, Duke University, Durham, NC; 3 Center for Child and Family Policy and the Sanford School of Public Policy, Duke University, Durham, NC

**Keywords:** adolescence, assault, neighborhood characteristics, psychosis, trauma, urbanicity

## Abstract

*Background:* Little is known about the impact of urbanicity, adverse neighborhood conditions and violent crime victimization on the emergence of adolescent psychotic experiences. *Methods:* Participants were from the Environmental Risk (E-Risk) Longitudinal Twin Study, a nationally-representative cohort of 2232 British twins who were interviewed about adolescent psychotic experiences at age 18. Urbanicity, neighborhood characteristics, and personal victimization by violent crime were measured during childhood and adolescence via geocoded census data, surveys of over 5000 immediate neighbors of the E-Risk participants, and interviews with participants themselves. *Results:* Adolescents raised in urban vs rural neighborhoods were significantly more likely to have psychotic experiences (OR = 1.67, 95% CI = 1.21–2.30, *P* = .002). This association remained significant after considering potential confounders including family socioeconomic status, family psychiatric history, and adolescent substance problems (OR = 1.43, 95% CI = 1.01–2.03, *P* = .042), but became nonsignificant after considering adverse social conditions in urban neighborhoods such as low social cohesion and high neighborhood disorder (OR = 1.35, 95% CI = 0.94–1.92, *P* = .102). The combined association of adverse neighborhood social conditions and personal crime victimization with adolescent psychotic experiences (adjusted OR = 4.86, 95% CI = 3.28–7.20, *P* < .001) was substantially greater than for either exposure alone, highlighting a potential interaction between neighborhood conditions and crime victimization (interaction contrast ratio = 1.81, 95% CI = −0.03 to 3.65) that was significant at the *P* = .054 level. *Conclusions:* Cumulative effects of adverse neighborhood social conditions and personal victimization by violent crime during upbringing partly explain why adolescents in urban settings are more likely to report psychotic experiences. Early intervention efforts for psychosis could be targeted towards victimized youth living in urban and socially adverse neighborhoods.

## Background

Up to 1 in 3 adolescents in the general population at some point experience subclinical psychotic phenomena such as attenuated forms of auditory hallucinations and paranoid delusions.^1–4^ Though relatively common, early psychotic experiences are associated with a greater adulthood risk for psychotic disorders and other psychiatric problems including substance abuse, depression, and suicidal behavior.^5–7^ Because early intervention offers the best hope for improving outcomes in psychosis^8^ and adult psychopathology more generally,^9^ it is crucial to understand how the wider structural and social environment may influence psychotic experiences among young people in order to design and effectively target preventive interventions.

To date, most prior research on the emergence of adolescent psychotic experiences has focused on individual-level risk factors^10^ and little is currently known about the potential impact of macro-level structures such as urbanicity and neighborhood-level social processes like social fragmentation and crime. These common forms of wider environmental exposures have been implicated in adult psychotic disorder,^11–21^ and adult psychosis shares similar social and behavioral risk factors as early psychotic phenomena.^10,22^ Early expressions of psychosis are more likely to persist and reach clinical significance among urban vs nonurban youth,^23,24^ but the reasons for this are unclear. We previously showed that adverse neighborhood social conditions in early childhood, such as low social cohesion and high crime, explained one quarter of the association between urbanicity and *childhood* psychotic symptoms.^25^ Elucidating the role of macro- and neighborhood-level exposures in *adolescent* psychotic experiences could be particularly informative for early-intervention efforts, because the clinical relevance of psychotic phenomena increases later in adolescence.^26^

Cities (vs rural settings) have higher rates of violent crime^27,28^ and tend to be more threatening^26^ and less socially cohesive.^25,29^ Additionally, 16–24 year-olds in the United Kingdom are 3 times more likely than other age groups to be victimized by a violent crime.^30^ Therefore, many adolescents raised in cities are not only embedded in more socially adverse neighborhoods, but are also more likely be personally victimized by crime compared to other age groups and peers living in rural neighborhoods. Given that cumulative trauma (ie, an accumulation of stressful exposures such as social adversity and victimization) is implicated in risk for psychosis,^31–34^ we hypothesized that one of the reasons that young people in urban settings are at increased risk for psychotic phenomena is that they experience a greater accumulation of neighborhood-level social adversity and personal experiences of violence during upbringing. No study has yet explored the potential cumulative effects of adverse neighborhood social conditions and personal crime victimization on the emergence of psychotic experiences during adolescence.

The present study addresses this topic with data from a nationally-representative cohort of over 2000 British adolescents, who have been interviewed repeatedly up to age 18, with comprehensive assessments of victimization and psychotic experiences and high-resolution measures of the built and social environment. We asked: (1) Are psychotic experiences more common among adolescents raised in urban vs rural settings? And does this association hold after controlling for neighborhood-level deprivation (ie, poverty), as well as individual- and family-level factors, that might otherwise explain the relationship? (2) Can the association between urban upbringing and adolescent psychotic experiences be explained by urban neighborhoods having lower levels of social cohesion and higher levels of neighborhood disorder (subsequently defined as exposure to *neighborhood social adversity*)? (3) Are psychotic experiences more common among adolescents who have been personally victimized by a violent crime? And (4) Is there a cumulative effect of neighborhood social adversity and personal crime victimization on adolescent psychotic experiences? In addition, the present study conducted sensitivity analyses using adolescent psychotic *symptoms* as the outcome (which are psychotic experiences verified by clinicians).

## Methods

### Study Cohort

Participants were members of the Environmental Risk (E-Risk) Longitudinal Twin Study, which tracks the development of a nationally-representative birth cohort of 2232 British twin children born in 1994–1995 and initially assessed in home visits at age 5. Follow-up home-visits were conducted when participants were aged 7, 10, 12, and 18 (participation rates were 98%, 96%, 96%, and 93%, respectively). At age 18, the E-Risk sample comprised 2066 participants. All but 3 participants completed the psychotic experiences interview at age 18. The final sample for this study was therefore 2063 individuals, comprising 55% monozygotic twin pairs and 48% males. There were no differences between those who did and did not take part at age 18 in terms of age-5 socioeconomic status (SES) (χ ^2^ = 0.86, *P* = .65), age-5 IQ scores (*t* = 0.98, *P* = .33), or age-5 internalizing or externalizing behavior problems (*t* = 0.40, *P* = .69 and *t* = 0.41, *P* = .68, respectively). The Joint South London and Maudsley and the Institute of Psychiatry Research Ethics Committee approved each phase of the study. Parents gave informed consent, and participants gave assent at ages 5–12 and informed consent at age 18. Full details about the sample are reported elsewhere,^35^ and in the supplementary materials.

### Measures

#### Adolescent Psychotic Phenomena.

The present study uses 2 measures of psychotic phenomena which were both obtained from private interviews when participants were aged 18.

Our primary outcome was a self-report measure of adolescent psychotic experiences which reflects the methodology used by many groups in the psychosis prodrome research field.^36^ At age 18, each E-Risk participant was privately interviewed by a research worker about 13 psychotic experiences occurring since age 12. Seven items pertained to delusions and hallucinations and this interview has been described in detail previously^22^ and in the supplementary materials. Six items pertained to unusual experiences which drew on item pools since formalized in prodromal psychosis instruments including the PRIME-screen and SIPS.^36^ These included “I worry that my food may be poisoned” and “My thinking is unusual or frightening.” Interviewers coded each item 0, 1, 2 indicating respectively “not present,” “probably present” and “definitely present.” All 13 items were summed to create a psychotic experiences scale (range = 0–18, *M* = 1.19, SD = 2.58). Scores were placed into an ordinal scale. Just over 30% of participants had at least 1 psychotic experience between ages 12 and 18: 69.8% reported no psychotic experiences (coded 0; *n* = 1440), 15.5% reported 1 or 2 psychotic experiences (coded 1; *n* = 319), 8.1% reported 3 to 5 psychotic experiences (coded 2; *n* = 166), and 6.7% reported 6 or more psychotic experiences (coded 3; *n* = 138). This is similar to the prevalence of self-reported psychotic experiences in other community samples of teenagers and young adults.^1–4^

We additionally examined clinically-verified adolescent psychotic symptoms as a secondary outcome, using the same methodology as used at age 12.^22^ Responses to the 7 hallucination/delusion items were verified by a team of clinicians, including child and adolescent psychiatrists, to capture more clinically pertinent psychotic symptoms. Full details on the verification procedure for adolescent psychotic symptoms are provided in the supplementary materials. At age 18, 2.9% (*N* = 59) of participants were designated as having experienced at least 1 definite psychotic symptom.

#### Urbanicity.

Urbanicity was defined based on data from the Office of National Statistics (ONS) Rural-Urban Definition for Small Area Geographies (RUC2011) classifications.^37^ The ONS classifications utilized 2011 census data. Detailed information on ONS’s creation of RUC2011 is available on the ONS webpages (https://www.gov.uk/government/uploads/system/uploads/attachment_data/file/239477/RUC11methodologypaperaug_28_Aug.pdf; this link was working on April 20, 2017) and in the supplementary materials. ONS urbanicity scores (range 1–10) were assigned to every E-Risk family via the family’s postcode when participants were aged 12. Given the low numbers within some rural categories, urbanicity was collapsed into 3 levels (1 = rural: all rural settings; 2 = intermediate: urban cities and towns; and 3 = urban: major and minor conurbations [conurbations are densely populated, large urban regions resulting from the expansion and coalescence of adjacent cities and towns]). E-Risk families are nationally-representative in terms of ONS urbanicity classifications; 32.2% of E-Risk children lived in urban settings at age 12 compared to 36.1% nationwide; 47.9% vs 45.0% lived in intermediate settings; and 19.9% vs 18.9% lived in rural settings.

#### Neighborhood Characteristics.

Social conditions (ie, social processes) in the participants’ neighborhoods were estimated via a postal survey sent to residents living alongside E-Risk families when participants were aged 13–14.^38,39^ Survey respondents, who were typically living on the same street or within the same apartment block as the participants in our study, reported on various characteristics of their immediate neighborhood, including levels of *social cohesion* and *neighborhood disorder.*^40,41^ Surveys were returned by an average of 5.18 (SD = 2.73) respondents per neighborhood, and there were at least 2 responses for 95% of neighborhoods (*N* = 5601 respondents). We were interested in social cohesion and neighborhood disorder because they collectively capture neighborhood characteristics that could plausibly influence risk for psychotic phenomena, such as trust and support between neighbors and physical and social signs of threat in the neighborhood. Social cohesion (5 items, each coded 0–4) was assessed by asking residents whether their neighbors shared values and trusted and got along with each other, etc. Neighborhood disorder (14 items, each coded 0–2) was assessed by asking residents whether certain problems affected their neighborhood, including muggings, assaults, vandalism, graffiti and deliberate damage to property, etc. Items within each neighborhood characteristic scale were averaged to create summary scores from each of the 5601 resident respondents. Neighborhood characteristic scores for each E-Risk family were then created by averaging the summary scores of respondents within that family’s neighborhood. The resulting variables approach normal distribution across the full potential range (Social cohesion: *M* = 2.23, SD = 0.50, range = 0–3.71; Neighborhood disorder: *M* = 0.49, SD = 0.34, range = 0–1.93). Supplementary table 1 demonstrates that urban neighborhoods were characterized by significantly lower levels of social cohesion and significantly higher levels of neighborhood disorder. Additionally, we indexed the most socially adverse neighborhoods by combining social cohesion with neighborhood disorder. Participants who had lived in neighborhoods that were simultaneously characterized by lower than average social cohesion and higher than average neighborhood disorder were designated as having experienced neighborhood social adversity (coded 1: 35.9% of participants, *N* = 772).

#### Personal Crime Victimization.

Personal experiences of violent crime victimization were assessed in private interviews with the participants at age 18 via the Juvenile Victimization Questionnaire 2nd revision (JVQ-R2)^42^ adapted as a clinical interview (see Fisher et al^43^ and supplementary materials for full details). JVQ crime victimization comprised 9 items, each enquiring about the period “since you were 12” (eg, “Did anyone hit or attack you on purpose with an object or weapon like a stick, rock, gun, knife or anything that hurt?”). The worst experience (according to the participant) was rated using a 6-point scale: 0 = not exposed, then 1–5 for increasing levels of severity, reflecting the level of physical harm that had occurred. In the present study, crime victimization was dichotomized to represent the most violent forms of crime where injury or threat to life was likely, with participants who reported the top 2 levels of JVQ crime victimization (levels 4/5) designated as having experienced *personal crime victimization* (coded 1: 19.3% of participants, *N* = 398).

#### Neighborhood-Level Deprivation.

Neighborhood-level deprivation was constructed using A Classification of Residential Neighborhoods (ACORN), a geodemographic discriminator developed by CACI Information Services (http://www.caci.co.uk/; this link was working on April 20, 2017). Detailed information about ACORN’s classification of neighborhood-level deprivation has been provided previously.^38,44,46^ Briefly, CACI utilized over 400 variables from 2001 census data for Great Britain (eg, educational qualifications, unemployment, housing tenure) and CACI’s consumer lifestyle database. Following hierarchical-cluster-analysis, CACI created 5 distinct and homogeneous ordinal groups ranging from “Wealthy Achiever” (coded 1) to “Hard Pressed” (coded 5) neighborhoods. Neighborhood-level deprivation scores for the E-Risk families were then created by identifying the ACORN classification for that family’s postcode when children were aged 12. E-Risk families are representative of UK households across the spectrum of neighborhood-level deprivation: 25.6% of E-Risk families live in “wealthy achiever” neighborhoods compared to 25.3% of households nation-wide; 5.3% vs 11.6% live in “urban prosperity” neighborhoods; 29.6% vs 26.9% live in “comfortably off” neighborhoods; 13.4% vs 13.9% live in “moderate means” neighborhoods; and 26.1% vs 20.7% live in “hard-pressed” neighborhoods.^45,46^

#### Family- and Individual-Level Covariates.

Family SES was measured via a composite of parental income, education, and occupation when participants were aged 5. The latent variable was categorized into tertiles (ie, low-, medium-, and high-SES). Family psychiatric history and maternal psychosis were both considered as proxy indicators of genetic and environmental risks, to control for potential social drift whereby individuals with mental illness may be more likely to move to adverse neighborhoods. Both were assessed when participants were aged 12. In private interviews, mothers reported on family history of DSM disorders,^47,48^ which was converted to a proportion (0–1.0) of family members with a history of psychiatric disorder. For maternal psychosis, mothers were interviewed using the Diagnostic Interview Schedule^49^ for DSM-IV^50^ which provides a symptom count for characteristic symptoms of schizophrenia (eg, hallucinations, delusions, anhedonia). Alcohol and cannabis dependence were considered because alcohol and cannabis are conceivably more available in cities, and abuse of these substances is associated with psychotic symptoms.^51,52^ We interviewed participants when they were aged 18 for the presence of alcohol/cannabis dependence according to DSM-IV criteria. Assessments were conducted in face-to-face interviews using the DIS.^51^ The rates were 12.8% (*N* = 263) and 4.3% (*N* = 89), respectively. Childhood psychotic symptoms at age 12 (described previously^22^) were included as a potential confound in models involving crime victimization because early psychotic phenomena have been associated with the likelihood of experiencing victimization. At age 12, 5.9% (*N* = 125) of children reported psychotic symptoms. Further details on the covariates are provided in the supplementary materials.

#### Adolescent Major Depression.

Specificity analyses were conducted with adolescent depression as the outcome, because psychotic experiences and depression commonly co-occur and share similar aetiology.^53^ Adolescent major depression was assessed at age 18 following DSM-IV criteria in face-to-face interviews using the DIS.^51^ By age 18, 20.1% (*N* = 414) of adolescents had met DSM-IV criteria for a major depressive episode.

### Statistical Analysis

We conducted analyses following 5 steps. First, logistic regression was used to test whether psychotic experiences (between ages 12 and 18) were more common among adolescents raised in urban neighborhoods. We controlled for family- and individual-level factors and for neighborhood-level deprivation to check that the association was not explained by these characteristics which could potentially differ between urban vs rural residents. We also examined the association between urbanicity and adolescent major depression to check for specificity of the previous findings. Second, because urban neighborhoods are characterized by lower levels of social cohesion and higher levels of neighborhood disorder (supplementary table 1) we tested whether levels of these neighborhood characteristics accounted for the association between urbanicity and adolescent psychotic experiences, and we also estimated the separate associations of social cohesion and neighborhood disorder with adolescent psychotic experiences. Third, using logistic regression we checked whether adolescents who had lived in the most socially adverse neighborhoods (neighborhood characterized by both low social cohesion and high neighborhood disorder) were more likely to be personally victimized by violent crime and, in turn, whether psychotic experiences were more common among adolescents who had been victimized. Fourth, using interaction contrast ratio analysis we investigated potential cumulative and interactive effects of adverse neighborhood social conditions and personal victimization by violent crime on adolescent psychotic experiences. Four exposure categories were created for this analysis by combining *neighborhood social adversity* with *personal crime victimization* (0 = not exposed to either; 1 = lived in the most adverse neighborhoods but not personally victimized by violent crime; 2 = personally victimized by violent crime but did not live in the most adverse neighborhoods; and 3 = exposed to both the most socially adverse neighborhood conditions and also personally victimized by violent crime). Finally, sensitivity analyses were conducted using the clinically-verified adolescent psychotic symptoms as the outcome measure. All analyses were conducted in STATA 14.2 (Stata-Corp), and accounted for the nonindependence of twin observations using the “CLUSTER” command. This procedure is derived from the Huber-White variance estimator, and provides robust standard errors adjusted for within-cluster correlated data.^54^ Note: ordinal logistic regression was used in analyses where adolescent psychotic experiences was the dependent variable, because this was on an ordinal (rather than binary) scale.

## Results

### Are Psychotic Experiences More Common Among Adolescents Raised in Urban vs Rural Neighborhoods?

Model 1 in table 1 shows that as the level of childhood urbanicity increased from rurality, odds for adolescent psychotic experiences also increased (intermediate urbanicity: OR = 1.37, 95% CI = 1.01–1.86, *P* = .042; highest urbanicity: OR = 1.67, 95% CI = 1.21–2.30, *P* = .002). Crucially, model 2 in table 1 highlights that the association between the most urban setting and adolescent psychotic experiences remained significant after considering a range of potential family- and individual-level confounders (family SES, family psychiatric history, maternal psychosis, and adolescent alcohol/cannabis dependence) and neighborhood-level deprivation (OR = 1.43, 95% CI = 1.01–2.03, *P* = .042), indicating that the association was not likely due to compositional effects. Moreover, the association also demonstrated a degree of specificity in that urban residency was not significantly associated with adolescent depression (unadjusted OR = 0.94, 95% CI = 0.68–1.31, *P* = .736).

**Table 1.** Association Between Childhood Urbanicity and Adolescent Psychotic Experiences

Model Specification	Level of Urbanicity^a^	Covariates	Association Between Childhood Urbanicity and Adolescent Psychotic Experiences^b^		
			OR	95% CI	*P* Value
Model 1	Rural		[Reference]	—	—
	Intermediate		1.37	1.01–1.86	.042
	Urban		1.67	1.21–2.30	.002
Model 2	Rural		[Reference]	—	—
	Intermediate		1.11	0.81–1.54	.513
	Urban		1.43	1.01–2.03	.042
		Family socioeconomic status	1.20	1.02–1.41	.029
		Family psychiatric history	1.99	1.30–3.06	.002
		Maternal psychotic symptoms	1.09	0.96–1.23	.187
		Adolescent alcohol dependence	2.20	1.66–2.92	<.001
		Adolescent cannabis dependence	4.21	2.60–6.82	<.001
		Neighborhood-level deprivation	1.10	1.00–1.20	.044
Model 3	Rural		[Reference]	—	—
	Intermediate		1.17	0.85–1.62	.329
	Urban		1.35	0.94–1.92	.103
		Neighborhood social conditions	1.28	1.11–1.48	.001

*Note*: OR, odds ratio from ordinal logistic regression.

^a^3-level urbanicity at age 12: Rural = rural towns and fringes, villages, hamlets, isolated dwellings; Intermediate = urban cities and towns; Urban = major and minor conurbations.

^b^The association of childhood urbanicity (and other covariates) with adolescent psychotic experiences was calculated with ordinal logistic regression because adolescent psychotic experiences are on an ordinal (0–3) rather than binary scale. Model 1—the unadjusted association between childhood urbanicity and adolescent psychotic experiences (sample size = 1978 participants). Model 2—adjusted for family-level characteristics (family socioeconomic status, family psychiatric history, maternal psychotic symptoms), individual-level characteristics (adolescent alcohol dependence and adolescent cannabis dependence), and neighborhood-level deprivation at age 12 (sample size = 1900 participants). Model 3—adjusted for neighborhood social conditions (social cohesion and neighborhood disorder) at age 12 (sample size = 1956 participants). Sample sizes vary slightly between models due to small numbers of participants missing data on independent variables. All analyses account for the nonindependence of twin observations.

### Can the Association Between Growing Up in an Urban (vs Rural) Setting and Adolescent Psychotic Experiences be Explained by Social Conditions of Urban Neighborhoods?

Model 3 in table 1 shows that after considering resident-reported neighborhood social conditions, the association between living in the most urban setting and adolescent psychotic experiences was attenuated to below conventional levels of significance (OR = 1.35, 95% CI = 0.94–1.92, *P* = .103). That is, almost half of the effect of urbanicity on adolescent psychotic experiences (mediatory estimates are supported by pathway analyses^55^) was explained by the levels of social cohesion and neighborhood disorder in urban vs rural neighborhoods. In table 2 we additionally show the independent effects of social cohesion and neighborhood disorder on adolescent psychotic experiences, with the neighborhood characteristic measures categorized at various thresholds. In short, psychotic experiences were more common among adolescents who had lived in neighborhoods with lower levels of social cohesion and higher levels of neighborhood disorder, and these associations were very similar regardless of the threshold used.

**Table 2.** Association Between Neighborhood Characteristics and Adolescent Psychotic Experiences With Neighborhood Characteristics Categorized at Various Thresholds

Neighborhood Characteristic	Association Between Neighborhood Characteristics and Adolescent Psychotic Experiences								
	Full-Scale Neighborhood Characteristics^a^			Neighborhood Characteristics Dichotomized at the Mean^b^			Neighborhood Characteristics Dichotomized at the Tertile^c^		
	OR	95% CI	*P* Value	OR	95% CI	*P* Value	OR	95% CI	*P* Value
Low social cohesion	1.57	1.26–1.95	<.001	1.53	1.24–1.89	<.001	1.54	1.23–1.93	<.001
High neighborhood disorder	2.07	1.52–2.81	<.001	1.73	1.40–2.14	<.001	1.53	1.23–1.91	<.001

*Note*: E-Risk, Environmental Risk; OR, odds ratio from ordinal logistic regression.

^a^Analyses were conducted using the full-scale neighborhood characteristic variables. That is, the average of resident-rated neighborhood characteristic scores for each E-Risk neighborhood. Social cohesion was reverse scored to facilitate comparison with neighborhood disorder.

^b^The full-scale neighborhood characteristic variables were dichotomized at the mean, so that low social cohesion was a score lower than the mean, and high neighborhood disorder was a score higher than the mean.

^c^The full-scale neighborhood characteristic variables were dichotomized at the tertile, so that low social cohesion was a score lower than the 33rd centile, and high neighborhood disorder was a score higher than the 66th centile. All analyses account for the nonindependence of twin observations.

### Are Psychotic Experiences More Common Among Adolescents Who Have Been Personally Victimized by a Violent Crime?

Among adolescents who had lived in the most socially adverse neighborhoods (neighborhoods that were simultaneously characterized by low social cohesion and high neighborhood disorder), 24.0% had been personally victimized by a violent crime compared to 15.1% of adolescents who had lived in more favorable neighborhood conditions (OR = 1.78, 95% CI = 1.32–2.41, *P* < .001). Furthermore, adolescents who had been victimized by violent crime had over 3 times greater odds of having psychotic experiences than non-victimized adolescents (OR = 3.76, 95% CI = 3.00–4.72, *P* < .001), and this association was not explained by the set of potential confounders reported in table 3 (OR = 2.90, 95% CI = 2.28–3.69, *P* < .001).

**Table 3.** The Cumulative Effect of Neighborhood Social Adversity and Personal Crime Victimization on Adolescent Psychotic Experiences

Exposure to Neighborhood Social Adversity and/or Personal Crime Victimization^a^	Association of Cumulative Exposure to Neighborhood Social Adversity and Personal Crime Victimization With Adolescent Psychotic Experiences^b^					
	Model 1			Model 2		
	OR	95% CI	*P* Value	OR	95% CI	*P* Value
0—Neither exposure	[Reference]	—	—	[Reference]	—	—
1—Neighborhood social adversity only	1.52	1.18–1.97	.001	1.33	1.00–1.78	.052
2—Personal crime victimization only	3.35	2.46–4.55	<.001	2.72	1.96–3.77	<.001
3—Neighborhood social adversity and personal crime victimization	6.79	4.81–9.60	<.001	4.86	3.28–7.20	<.001
Interaction between neighborhood social adversity and personal crime victimization	ICR 2.92, 95% CI = 0.63–5.22, *P* = .013			ICR = 1.81, 95% CI = -0.03–3.65, *P* = .054		

*Note*: ICR, interaction contrast ratio; OR, odds ratio from ordinal logistic regression.

^a^These 4 exposure categories were created by combining neighborhood social adversity (neighborhood was simultaneously characterized by low social cohesion and high neighborhood disorder) with personal crime victimization: 0 = not exposed to either; 1 = lived in the most socially adverse neighborhood but not personally victimized by violent crime; 2 = personally victimized by violent crime but did not live in the most socially adverse neighborhood; and 3 = exposed to both the most socially adverse neighborhood conditions and also personally victimized by violent crime.

^b^The association of cumulative exposures to neighborhood social adversity and personal crime victimization with adolescent psychotic experiences was calculated with ordinal logistic regression because adolescent psychotic experiences are on an ordinal (0–3) rather than binary scale. Model 1—the unadjusted associations of neighborhood social adversity and personal crime victimization with adolescent psychotic experiences. Model 2—adjusted simultaneously for childhood psychotic symptoms, family-level characteristics (family socioeconomic status, family psychiatric history, maternal psychotic symptoms), individual-level characteristics (adolescent alcohol dependence and adolescent cannabis dependence), and neighborhood-level deprivation at age 12. All analyses account for the nonindependence of twin observations.

### Is There a Cumulative Effect of Neighborhood Social Adversity and Personal Crime Victimization on Adolescent Psychotic Experiences?

Given previous evidence that risk for psychosis increases incrementally following an accumulation of stressful exposures, we tested for cumulative and interactive effects of adverse neighborhood social conditions and personal crime victimization during upbringing on adolescent psychotic experiences. Table 3 shows that both neighborhood social adversity and personal crime victimization each had significant independent associations with adolescent psychotic experiences. However, focusing on model 2, which adjusts for all potential confounders, the combined effect of neighborhood social adversity and personal crime victimization on adolescent psychotic experiences was much greater than either exposure alone, at nearly 5 times the odds compared to unexposed adolescents (OR = 4.86, 95% CI = 3.28–7.20, *P* < .001). The interaction between neighborhood social adversity and personal crime victimization (ICR = 1.81, 95% CI = -0.03–3.65) was significant at the *P* = .054 level. That is, the odds for adolescent psychotic experiences among individuals who were exposed to both neighborhood social adversity and crime victimization was 1.81 points higher than the summed effects of the individual exposures (model 2 in table 3).

### Sensitivity Check: Are Urbanicity, Neighborhood Social Conditions, and Crime Victimization Also Associated With Adolescent Psychotic Symptoms (vs Experiences)?

Only 2.9% (*n* = 59) of adolescents met criteria for the clinically-verified psychotic symptoms. Adjusted model 2 in supplementary table 2 shows that participants raised in urban (vs rural) settings appeared to be at elevated risk for experiencing adolescent psychotic *symptoms*, though this association was nonsignificant (OR = 1.40, 95% CI = 0.57–3.41, *P* = .460). While the point estimate was very similar to that produced for adolescent psychotic *experiences* (OR = 1.43, 95% CI = 1.01–2.03, *P* = .042), the low base rate of verified symptoms in the current sample restricted our power to detect associations at this level. In addition, model 3 in supplementary table 2 revealed that neighborhood social adversity explained a similar proportion of the effect of the most urban residency on adolescent psychotic symptoms to that found for adolescent psychotic experiences. Finally, supplementary table 3 yielded very similar point estimates for the cumulative exposures categories, though some associations failed to reach statistical significance.

## Discussion

This study investigated the role of urbanicity, neighborhood social conditions, and personal crime victimization in adolescent psychotic experiences and revealed 3 initial findings. First, the association between growing up in an urban environment and adolescent psychotic experiences remained after considering a range of potential confounders including family SES, family psychiatric history, maternal psychosis, adolescent substance problems, and neighborhood-level deprivation. This association between urbanicity and psychotic experiences was explained, in part, by 2 features of the neighborhood social environment, namely lower levels of social cohesion and higher levels of neighborhood disorder. Second, personal victimization by violent crime was nearly twice as common among adolescents in the most socially adverse neighborhoods, and adolescents who had experienced such victimization had over 3 times greater odds of having psychotic experiences. Third, the cumulative effect of neighborhood social adversity and personal crime victimization on adolescent psychotic experiences was substantially greater than either exposure alone, highlighting a potential interaction between these exposures. That is, adolescents who had lived in the most adverse neighborhood conditions and been personally victimized were at the greatest risk for psychotic experiences during adolescence.

The present findings extend previous evidence from this cohort implicating childhood urbanicity and neighborhood characteristics in the occurrence of childhood psychotic symptoms.^25^ Here we show that the effects of urban and socially adverse neighborhood conditions on psychotic experiences are not limited to childhood, but continue into adolescence when psychotic phenomena become more clinically relevant.^26^ These findings support previous evidence demonstrating higher rates of psychosis-proneness and prodromal status among adolescents and young adults in urban,^1^ threatening,^56^ and socially fragmented neighborhoods.^57^ Late adolescence heralds the peak age at which psychotic disorders are typically diagnosed.^58^ If a degree of aetiological continuity truly exists between adolescent psychotic experiences and adult psychotic disorder, ours and other recent findings tentatively support a mechanism linking adverse neighborhood conditions during upbringing with psychosis in adulthood.

In our study, the combined effect of adverse neighborhood social conditions and personal victimization by violent crime was greater than the independent effects of each. This is consistent with cumulative stress models and previous studies showing that risk for psychosis phenotypes increases as the frequency and severity of stressful exposures increase.^31–34,59,60^ Several biological and psychological mechanisms could explain why adolescents who were exposed to neighborhood social adversity and violent crime during upbringing were more prone to psychotic experiences. Prolonged and acute early-life stress is purported to dysregulate the biological stress response^61–63^ and lead to dopaminergic sensitization, which is the leading hypothesized neurochemical pathway for the positive symptoms of psychosis.^61,64^ In addition, adolescents who grow up in threatening neighborhoods with weak or absent community networks could develop psychosis-like cognitive schemas such as paranoia, hypervigilance, and negative attributional styles.^65,66^ A cognitive pathway (rather than a nonspecific stress mechanism alone) could explain why effects were apparent for psychotic experiences but not major depression. Our findings tentatively suggest a mechanism whereby childhood exposure to neighborhood social adversity sensitizes individuals to subsequent stressful experiences such as crime victimization. This hypothesized mechanism is supported by recent evidence of neurological differences in social stress reactivity between adults with urban vs rural upbringing.^67,68^ Further research into the influence of neighborhood exposures on childhood neurocognitive development could shed light on this hypothesized mechanism.

### Limitations

Several limitations should be considered. First, causality of findings from this observational study cannot be assumed. Noncausal mechanisms, such as the selection of genetically high-risk families into urban and adverse neighborhoods, remain possible,^69^ though our findings were not explained by proxy indicators of genetic and familial risk. Second, neighborhood conditions were measured approximately 5 years before adolescent psychotic experiences were assessed. However, the vast majority of adolescents (71.4%, *n* = 1475) reported that they did not move house between ages 12 and 18. Third, though crime victimization was more common in adverse neighborhoods, we do not know the extent to which these victimization experiences occurred outside the home. Perpetrators of physical violence are often family members,^70^ suggesting that our measure of violent crime captured victimization inside as well as outside the home. Fourth, psychotic experiences are associated with adult psychosis but also with other serious psychiatric conditions^5^; while a degree of specificity was suggested in that the effect of urbanicity on psychotic experiences was not replicated for adolescent depression and was not explained by adolescent substance problems, it is probable that the mental health implications of growing up in an urban setting extend beyond psychosis.^71^ In addition, associations arising for the clinically-verified psychotic symptoms were often nonsignificant. It is possible that the low prevalence of psychotic symptoms in this sample restricted our power to detect associations. However, it is also possible that the self-report measure of adolescent psychotic experiences captured genuine experiences (eg, being followed by a stranger) as well as psychotic phenomena (eg, being followed by a detective). This may have inflated the associations arising for adolescent psychotic experiences, though it is reassuring that point estimates were fairly similar to those produced for psychotic symptoms. Finally, our findings come from a sample of twins which potentially differ from singletons. However, E-Risk families closely match the distribution of UK families across the spectrum of urbanicity^38^ and neighborhood-level deprivation.^46^ Furthermore, the prevalence of adolescent psychotic experiences among E-Risk participants is similar to non-twin samples of adolescents and young adults.^1–4^

## Conclusions

Our findings provide initial evidence that adverse neighborhood social conditions and violent crime victimization, which are relatively common exposures particularly among urban youth, increase risk for adolescent psychotic experiences. From a public health perspective, ours and other recent findings on geospatial correlates of early psychosis phenotypes^56,57^ suggest that preventative early intervention strategies for psychosis might capture particularly high-risk groups if targeted towards youth living in urban and socially adverse neighborhoods. As increasing numbers of youth around the world are living in cities,^72^ there is a growing need to improve our understanding of how both built and social features of urban settings are supporting and challenging young people’s mental health.

## Supplementary Material

Supplementary material is available at *Schizophrenia Bulletin* online.

## Funding

The E-Risk Study is funded by the Medical Research Council (G1002190). Additional support was provided by National Institute of Child Health and Human Development (HD077482); Google Streetview; British Academy (SQ140024); and the Jacobs Foundation. J.N. received a Multidisciplinary Studentship from the Economic and Social Research Council (ESRC). L.A. is the Mental Health Leadership Fellow for the UK ESRC. H.L.F. is supported by an MQ Fellows Award (MQ14F40). C.L.O. is a Jacobs Foundation Advanced Research Fellow and a Canadian Institute for Advanced Research Fellow.

## Supplementary Material

Supplementary Materials
