# Supplementary material for: Cumulative Effects of Neighborhood Social Adversity and Personal Crime Victimization on Adolescent Psychotic Experiences
Source: Schizophr Bull. 2017 May 22;44(2):348–58. doi: 10.1093/schbul/sbx060 (PMC5815129; doi:10.1093/schbul/sbx060)
Supplement: Supplementary Materials [file sbx060_suppl_supplementary_materials.doc]

**Supplementary Materials**

**Supplementary Methods**

*Study Cohort*

Participants were members of the Environmental Risk (E-Risk) Longitudinal Twin Study, which tracks the development of a nationally-representative birth cohort of 2232 British twin children. The sample was drawn from a larger cohort of twins born in England and Wales in 1994-1995.[^1^](#_ENREF_1) Full details about the sample are reported elsewhere.[^2^](#_ENREF_2) Briefly, the E-Risk sample was constructed in 1999-2000, when 1116 families with same-sex 5-year-old twins (93% of those eligible) participated in home-visit assessments. Families were recruited to represent the UK population of families with newborns in the 1990s, based on residential location throughout England and Wales and mothers’ age. Teenaged mothers with twins were over-selected to replace high-risk families who were selectively lost to the register through non-response. Older mothers having twins via assisted reproduction were under-selected to avoid an excess of well-educated older mothers. E-Risk families are representative of UK households across the spectrum of neighborhood-level deprivation: 25.6% of E-Risk families live in “wealthy achiever” neighborhoods compared to 25.3% of households nation-wide; 5.3% vs 11.6% live in “urban prosperity” neighborhoods; 29.6% vs 26.9% live in “comfortably off” neighborhoods; 13.4% vs 13.9% live in “moderate means” neighborhoods; and 26.1% vs 20.7% live in “hard-pressed” neighborhoods. E-Risk families under-represent “urban prosperity” neighborhoods because such households are likely to be childless. The sample comprised 56% monozygotic and 44% dizygotic twin pairs, and sex was evenly distributed within zygosity (49% male). All families were English speaking, and the majority (93.7%) were White.

Follow-up home visits were conducted when children were 7 years (98% of the 1116 E-Risk Study families participated), 10 years (96% participation), 12 years (96% participation), and, most recently in 2012–2014, 18 years (93% participation). Home visits at ages 5, 7, 10, and 12 years included assessments with participants as well as their mother (or primary caretaker); the home visit at age 18 included interviews only with the participants. Each twin participant was assessed by a different interviewer. The average age of the twins at the time of the age 18 assessment was 18.4 years (*SD*=0.36); all interviews were conducted after the 18^th^ birthday. There were no differences between those who did and did not take part at age 18 in terms of socioeconomic status (SES) assessed when the cohort was initially defined (χ^2^=0.86, *p*=0.65), age-5 IQ scores (*t*=0.98, *p*=0.33), or age-5 internalizing or externalizing behavior problems (*t*=0.40, *p*=0.69 and *t*=0.41, *p*=0.68, respectively). The Joint South London and Maudsley and the Institute of Psychiatry Research Ethics Committee approved each phase of the study. Parents gave informed consent, and participants gave assent at ages 5-12 and informed consent at age 18.

*Measure of clinically-verified psychotic symptoms*

E-Risk Study members were visited by mental health trainees or professionals when they were aged 18.[^5^](#_ENREF_5) Each adolescent was privately interviewed about whether they had experienced 7 psychotic symptoms pertaining to delusions and hallucinations since age 12, with items including “have other people read your thoughts?,” “have you thought you were being followed or spied on?,” and “have you heard voices that other people cannot hear?.” This interview has been described in detail previously,^5^ and was used at age 12 to estimate childhood psychotic symptoms. Analyses involving crime victimization in the present study are adjusted for childhood psychotic symptoms. The item choice was guided by the Dunedin Study’s age-11 interview protocol[^6^](#_ENREF_6) and an instrument prepared for the Avon Longitudinal Study of Parents and Children.[^7^](#_ENREF_7) At age 18 (and 12) Interviewers coded each experience 0, 1, 2 indicating respectively “not a symptom,” “probable symptom,” and “definite symptom.” A conservative approach was taken in designating an adolescent’s report as a symptom. First, the interviewer probed using standard prompts designed to discriminate between experiences that were plausible (e.g., “I was followed by a man after school”) and potential symptoms (e.g., “I was followed by an angel who guards my spirit”), and wrote down the adolescent’s narrative description of the experience. Second, items and interviewer notes were assessed by psychologists’ expert in assessing psychosis, and a child and adolescent psychiatrist to verify the validity of the symptoms. Third, because Study members were twins, experiences limited to the twin relationship (e.g., “My twin and I often know what each other are thinking”) were coded as “not a symptom”. Adolescents were only designated as having psychotic symptoms if they reported at least one definite and verified symptom. At age 18, 2.9% (N=59) of adolescents reported having psychotic symptoms since age 12. This is somewhat lower than the prevalence of psychotic symptoms in this sample at age 12 (5.9%, N=125), consistent with the attenuation of psychotic symptoms documented from childhood to adulthood. Furthermore, our psychotic symptom measure has good construct validity, sharing many of the genetic, social, neurodevelopmental, and behavioural risks factors and correlates as adult schizophrenia.[^5^](#_ENREF_5)

*Personal crime victimization*

Personal experiences of violent crime victimization were assessed at age 18 via the Juvenile Victimization Questionnaire 2^nd^ revision (JVQ-R2);[^10^](#_ENREF_10) adapted as a clinical interview (see [Fisher et a^l.11^](#_ENREF_11) for full details). Participants were interviewed face-to-face with a modified version of the JVQ-R2 about exposure to a range of adverse experiences occurring during adolescence. JVQ crime victimization comprised nine items, each enquiring about the period “since you were 12” (e.g., “Did anyone try to kidnap you?”, “Did anyone hit or attack you on purpose with an object or weapon like a stick, rock, gun, knife or anything that hurt?”). Participants responded “yes” or “no” as to whether each type of victimization had occurred, and follow-up questions were asked concerning details of the worst event the participant had experienced. All information from the modified JVQ-R2 interview was compiled into victimization dossiers and rated by an expert in victimology (Dr. Helen Fisher) and three other trained E-Risk team members. Ratings were made using a 6-point scale: 0=not exposed, then 1–5 for increasing levels of severity, adapted from the standardized coding system used for the Childhood Experience of Care and Abuse interview (CECA). High levels of interrater reliability were achieved for the crime victimization severity ratings (intraclass correlation coefficient=0.89, p<0.001). The severity of crime victimization reflected the level of physical harm that had occurred. In the present study, crime victimization was dichotomized to represent very violent forms of crime, with adolescents who reported the top two levels of JVQ crime victimization (levels 4/5; injury or threat to life likely), designated as having experienced *personal crime victimization* (19.3%, N=398).

*Urbanicity*

Urbanicity was derived from the Office of National Statistics (ONS) Rural-Urban Definition for Small Area Geographies (RUC2011) classifications.[^14^](#_ENREF_14) The ONS classifications utilised 2011 census data, and were designed for application to small geostatistical units (e.g. Output Areas). Detailed information on ONS’s creation of RUC2011 is available on the ONS webpages (<https://www.gov.uk/government/uploads/system/uploads/attachment_data/file/239477/RUC11methodologypaperaug_28_Aug.pdf>). Briefly, RUC2011 was created by laying a grid of hectare cells (100m^2^) over England and Wales. Postcode addresses were assigned to cells, and residential densities were then calculated for increasing radii around each cell, providing each residential property with a “density profile”. This was combined with Output Area and contextual data, allowing each settlement to be assigned to one of ten urbanicity categories (Rural categories: sparse/non-sparse hamlets and isolated dwellings, sparse/non-sparse villages, sparse/non-sparse rural town and fringe; Urban categories: sparse/non-sparse city and town, and minor/major conurbations). ONS urbanicity scores were then assigned to every E-Risk family via the family’s postcode when children were aged 5, 7, 10 and 12. E-Risk families are highly representative of the UK in terms of ONS urbanicity classifications. For example, 27.9% of E-Risk children lived in major conurbations at age 12 compared to 32.6% nationwide; and 3.1% versus 3.3%, respectively, lived in hamlets and isolated dwellings. Given the low numbers within some rural categories, urbanicity was collapsed into three levels (1=all rural categories [19.9% of children at age 12]; 2=urban cities and towns [47.9% of children at age 12]; and 3=minor/major conurbations [32.2% of children at age 12]). (Note: the present studies’ urbanicity measure differs from that previously used by E-Risk investigators, in which over 5000 immediate neighbors of E-Risk families were surveyed about whether their neighborhood was in a city, town, suburb, village or the countryside. We opted to use the newly available ONS measure because it provides a more objective and precise means of classifying local areas. However, the ONS and resident survey measures overlap considerably in their urbanicity estimates: 94.1% of neighborhoods rated as urban by residents are urban according to the ONS, and 71.7% of neighborhoods rated as rural by residents are rural according to the ONS).

*Neighborhood characteristics*

Threatening characteristics in the participants’ neighborhoods were estimated via a postal survey sent to residents living alongside E-Risk families when children were aged 12. Questionnaires were sent to every household in the same postcode as the E-Risk families, excluding the E-Risk families themselves (addresses were identified from electoral roll records). The number of surveys sent ranged from 15 to 50 residences per neighborhood (Average=18.96, SE=0.21). Excluding undelivered surveys (N=600), the overall response rate was 28.1% (5601/19926). Survey respondents typically lived on the same street or within the same apartment block as the children in our study. Surveys were returned by an average of 5.18 (SD=2.73) respondents per neighborhood (range=0–18 respondents), and there were at least two responses from 95% of the neighborhoods (N=5,601 respondents).[^17^](#_ENREF_17)

*Family socioeconomic status*.

Family socioeconomic status (SES) was measured via a composite of parental income, parental education, and parental occupation. Parental income was measured as the entire income of the household. Parental education was the highest level of education achieved by either the mother or father (highest value taken), ranging from 1 (CSE [1], O Level [A-C], GCSE [A-C]) to 7 (Postgraduate degree). Parental occupation was the highest level of parental occupation of either parent, ranging from 1 (both parents unemployed [coded 2 if single unemployed mother]) to 9 (professional). The three SES indicators were highly correlated (*r*s = 0.57 – 0.67, all *p*’s<0.05) and loaded significantly onto one latent factor (*M* = 2.00, *SD* = 0.82; factor loadings = 0.80, 0.70 and 0.83 for parental income, education and occupation, respectively). These variables were then standardized and summed, before categorising into tertiles at the 33.33^rd^ and 66.66^th^ centile (low-, medium-, and high-SES).

**References**

**1.** Trouton A, Spinath FM, Plomin R. Twins early development study (TEDS): a multivariate, longitudinal genetic investigation of language, cognition and behavior problems in childhood. *Twin Res.* 2002;5:444-448.

**2.** Moffitt TE, The E-Risk Study Team. Teen‐aged mothers in contemporary Britain. *J Child Psychol Psychiatry.* 2002;43:727-742.

**3.** CACI Information Services. *ACORN user guide*. London: CACI; 2006.

**4.** Caspi A, Taylor A, Moffitt TE, Plomin R. Neighborhood deprivation affects children's mental health: Environmental risks identified in a genetic design. *Psychol Sci.* 2000;11:338-342.

**5.** Polanczyk G, Moffitt TE, Arseneault L, et al. Etiological and clinical features of childhood psychotic symptoms: Results from a birth cohort. *Arch Gen Psychiatry.* 2010;67:328-338.

**6.** Poulton R, Caspi A, Moffitt TE, et al. Children's self-reported psychotic symptoms and adult schizophreniform disorder: a 15-year longitudinal study. *Arch Gen Psychiatry.* 2000;57:1053-1058.

**7.** Schreier A, Wolke D, Thomas K, et al. Prospective study of peer victimization in childhood and psychotic symptoms in a nonclinical population at age 12 years. *Arch Gen Psychiatry.* 2009;66:527-536.

**8.** Kelleher I, Keeley H, Corcoran P, et al. Clinicopathological significance of psychotic experiences in non-psychotic young people: evidence from four population-based studies. *Br J Psychiatry.* 2012;201:26-32.

**9.** Zammit S, Kounali D, Cannon M, et al. Psychotic experiences and psychotic disorders at age 18 in relation to psychotic experiences at age 12 in a longitudinal population-based cohort study. *Am J Psychiatry.* 2013;170:742-750.

**10.** Finkelhor D, Hamby S, Turner H, ORMOD R. *The Juvenile Victimization Questionnaire: 2nd Revision (JVQ-R2)*: Durham, Crimes Against Children Research Center; 2011.

**11.** Fisher HL, Caspi A, Moffitt TE, et al. Measuring adolescents' exposure to victimization: the Environmental Risk (E-Risk) Longitudinal Twin Study. *Dev Psychopathol.* 2015;27:1399-1416.

**12.** Bifulco A, Brown GW, Harris TO. Childhood Experience of Care and Abuse (CECA): a retrospective interview measure. *Journal of Child Psychology and Psychiatry.* 1994;35:1419-1435.

**13.** Bifulco A, Brown G, Neubauer A, Moran P, Harris T. Childhood Experience of Care and Abuse (CECA): Training Manual. *London: Royal Halloway, University of London.* 1994.

**14.** Office for National Statistics. *Urban and rural area definitions for policy purposes in England and Wales: Methodology (v1.0)*. London: Office of National Statistics; 2013.

**15.** Newbury J, Arseneault L, Caspi A, et al. Why are children in urban neighborhoods at increased risk for psychotic symptoms? Findings from a UK longitudinal cohort study. *Schizophr Bull.* 2016;42(6):1372-1383.

**16.** Odgers CL, Moffitt TE, Tach LM, et al. The protective effects of neighborhood collective efficacy on British children growing up in deprivation: a developmental analysis. *Dev Psychol.* 2009;45:942-957.

**17.** Odgers CL, Caspi A, Bates CJ, Sampson RJ, Moffitt TE. Systematic social observation of children's neighborhoods using Google Street View: a reliable and cost-effective method. *J Child Psychol Psychiatry.* 2012;53:1009-1017.

***Supplementary Table 1.*** *Levels of social cohesion and neighborhood disorder according to level of urbanicity*

| **Level of urbanicity** | **Levels of social cohesion and neighborhood disorder according to level of urbanicity** | | | | |
| --- | --- | --- | --- | --- | --- |
|  | Social cohesion | |  | Neighborhood disorder | |
|  | M | SD |  | M | SD |
| Rural | 2.58 | 0.42 |  | 0.27 | 0.22 |
| Intermediate | 2.19 | 0.45 |  | 0.49 | 0.32 |
| Urban | 2.07 | 0.52 |  | 0.62 | 0.37 |
| Association between urbanicity and neighborhood characteristics | B = -0.24, (95% CI = -0.28 – -0.20), p < 0.001, *B* = -0.34 | |  | B = 0.17, (95% CI = 0.14 – 0.19), p < 0.001, *B* = 0.35 | |
|  |  | |  |  | |

Note: B, unstandardized beta coefficient; *B,* standardized beta coefficient; CI, confidence; M mean; SD, standard deviation. ^a^ Three-level urbanicity at age 12: Rural = rural towns and fringes, villages, hamlets, isolated dwellings; Intermediate = urban cities and towns; Urban = major and minor conurbations. The standardized (*B*) beta coefficients indicate the unit standard deviation change in each neighborhood characteristic given one unit standard deviation change in urbanicity, and allow comparison between social cohesion and neighborhood disorder which are on different scales. Standardized betas provide exactly the same point estimates as correlation coefficients and may be interpreted as correlations, with a score of +1.0 indicating a 100% positive correlation. Unstandardized beta (B) coefficients account for the non-independence of twin observations.

**Supplementary Table 2.** *Association between childhood urbanicity and adolescent psychotic symptoms.*

| **Model specification** | **Level of urbanicity ^a^** | **Covariates** | **Association between childhood urbanicity and adolescent psychotic symptoms** | | |
| --- | --- | --- | --- | --- | --- |
|  |  |  | OR | 95% CI | P value |
|  |  |  |  |  |  |
| Model 1 | Rural |  | [reference] |  | - |
|  | Intermediate |  | 1.44 | 0.64 – 3.22 | 0.373 |
|  | Urban |  | 1.68 | 0.73 – 3.85 | 0.218 |
|  |  |  |  |  |  |
|  |  |  |  |  |  |
| Model 2 | Rural |  | [reference] |  |  |
|  | Intermediate |  | 1.06 | 0.43 – 2.58 | 0.905 |
|  | Urban |  | 1.40 | 0.57 – 3.41 | 0.460 |
|  |  |  |  |  |  |
|  |  | Family socioeconomic status | 1.08 | 0.70 – 1.65 | 0.734 |
|  |  | Family psychiatric history | 2.51 | 0.73 – 8.66 | 0.146 |
|  |  | Maternal psychotic symptoms | 1.22 | 0.95 – 1.58 | 0.124 |
|  |  | Adolescent alcohol dependence | 2.14 | 1.11 – 4.14 | 0.023 |
|  |  | Adolescent cannabis dependence | 2.06 | 0.79 – 5.34 | 0.137 |
|  |  | Neighborhood-level deprivation | 1.14 | 0.88 – 1.47 | 0.312 |
|  |  |  |  |  |  |
|  |  |  |  |  |  |
| Model 3 | Rural |  | [reference] | - | - |
|  | Intermediate |  | 1.33 | 0.56 – 3.16 | 0.516 |
|  | Urban |  | 1.41 | 0.58 – 3.44 | 0.446 |
|  |  |  |  |  |  |
|  |  | Neighborhood social conditions | 1.34 | 0.99 – 1.80 | 0.055 |
|  |  |  |  |  |  |

Note: CI, confidence interval; OR, odds ratio from logistic regression. ^a^ Three-level urbanicity at age 12: Rural = rural towns and fringes, villages, hamlets, isolated dwellings; Intermediate = urban cities and towns; Urban = major and minor conurbations. Model 1 – the unadjusted association between childhood urbanicity and adolescent psychotic symptoms (Sample size = 1978). Model 2 – adjusted for family-level characteristics (family socioeconomic status, family psychiatric history, maternal psychotic symptoms), individual-level characteristics (adolescent alcohol dependence and adolescent cannabis dependence), and neighborhood-level deprivation at age 12 (sample size = 1900 participants). Model 3 – adjusted for neighborhood social conditions (social cohesion and neighborhood disorder) at age 12 (sample size = 1956 participants). Sample sizes vary slightly between models due to small numbers of participants missing data on independent variables. All analyses account for the non-independence of twin observations.

***Supplementary Table 3.*** The cumulative effect of neighborhood social adversity and personal crime victimization on adolescent psychotic symptoms

| **Exposure to neighborhood social adversity and/or personal crime victimization ^a^** | **Association of cumulative exposure to neighborhood social adversity and personal crime victimization with adolescent psychotic symptoms** | | | | | | |
| --- | --- | --- | --- | --- | --- | --- | --- |
|  | Model 1 | | |  | Model 2 | | |
|  | OR | 95% CI | P value |  | OR | 95% CI | P value |
| 0 – Neither exposure | [reference] | - | - |  | [reference] | - | - |
| 1 – Neighborhood social adversity only | 1.46 | 0.72 – 2.96 | 0.300 |  | 1.29 | 0.60 – 2.79 | 0.509 |
| 2 – Personal crime victimization only | 2.95 | 1.38 – 6.28 | 0.005 |  | 2.11 | 0.90 – 4.92 | 0.084 |
| 3 – Neighborhood social adversity and personal crime victimization | 4.52 | 2.18 – 9.36 | <0.001 |  | 3.19 | 1.34 – 7.57 | 0.009 |
|  |  |  |  |  |  |  |  |
| Interaction between neighborhood social adversity and personal crime victimization | ICR = 1.12, 95% CI = -1.96 – 4.19, p = 0.477 | | |  | ICR = 0.79, 95% CI = -1.74 - 3.31, p = 0.541 | | |
|  |  | | |  |  | | |

Note: CI, confidence interval; ICR, interaction contrast ratio; OR, odds ratio from logistic regression. ^a^ These four exposure categories were created by combining neighborhood social adversity (neighborhood was simultaneously characterized by low social cohesion and high neighborhood disorder) with personal crime victimization: 0=not exposed to either; 1=lived in the most socially adverse neighborhood but not personally victimized by violent crime; 2=personally victimized by violent crime but did not live in the most socially adverse neighborhood conditions; and 3=exposed to both the most socially adverse neighborhood conditions and also personally victimized by violent crime. Model 1 – the unadjusted associations of neighborhood social adversity and personal crime victimization with adolescent psychotic symptoms. Model 2 – adjusted simultaneously for childhood psychotic symptoms, family SES, family psychiatric history, maternal psychosis, adolescent cannabis dependence, adolescent alcohol dependence, and neighborhood-level deprivation. All analyses account for the non-independence of twin observations.
